# A rare pericallosal lipoma presenting with Lennox–Gastaut syndrome and a remarkable response to valproic acid

**DOI:** 10.1002/epd2.70249

**Published:** 2026-04-24

**Authors:** Rainier Mark Loidor L. Rapal, Hok Leong Chin, Douglas Nordli

**Affiliations:** ^1^ Department of Pediatric Neurology University of Chicago Chicago Illinois USA

We present a clinical vignette of a 4‐year‐old boy with epilepsy diagnosed at age 2, who developed a significant increase in seizure burden over a 12‐month period, with semiologies that included facial twitching, spasm of upper extremities, myoclonic body jerks, mutism, drooling, along with frequent falls and urinary incontinence.

Magnetic resonance imaging (MRI) revealed a lobulated posterior pericallosal lipoma (2.2 × 2.8 × 2.9 cm^3^) with global cerebral volume loss and splaying of the posterior medial thalami (Figure 1). Electroencephalography (EEG) demonstrated a pattern consistent with LGS, including myoclonic, tonic‐myoclonic, spasm‐tonic, and atypical absence seizures, along with abundant multifocal epileptiform discharges and generalized paroxysmal fast activity during sleep. After limited response to levetiracetam, pulse corticosteroids, and clobazam, valproic acid monotherapy was initiated, resulting in complete clinical seizure resolution and normalization of the EEG. Follow‐up EEG showed resolution of the previously noted high‐voltage disorganized background and epileptiform discharges (Figure 2).

**FIGURE 1:**
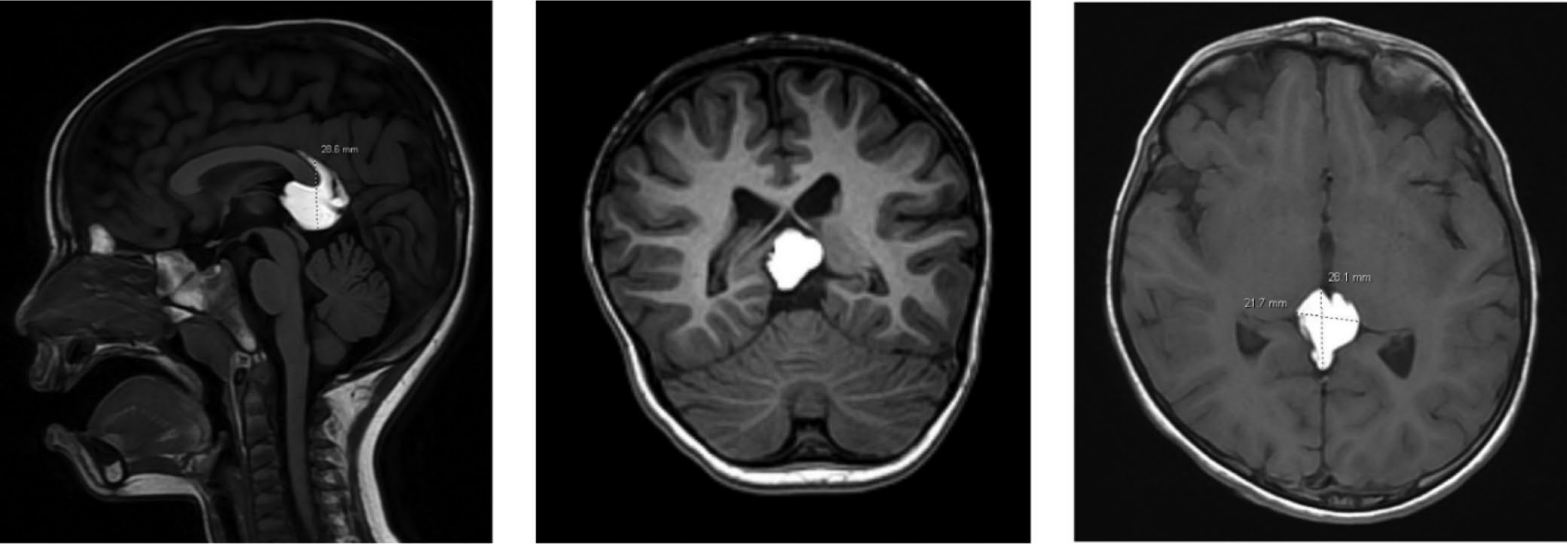
T1‐weighted sagittal, coronal, and axial MRI images illustrating the size and anatomical location of the intracranial lipoma.

**FIGURE 2:**
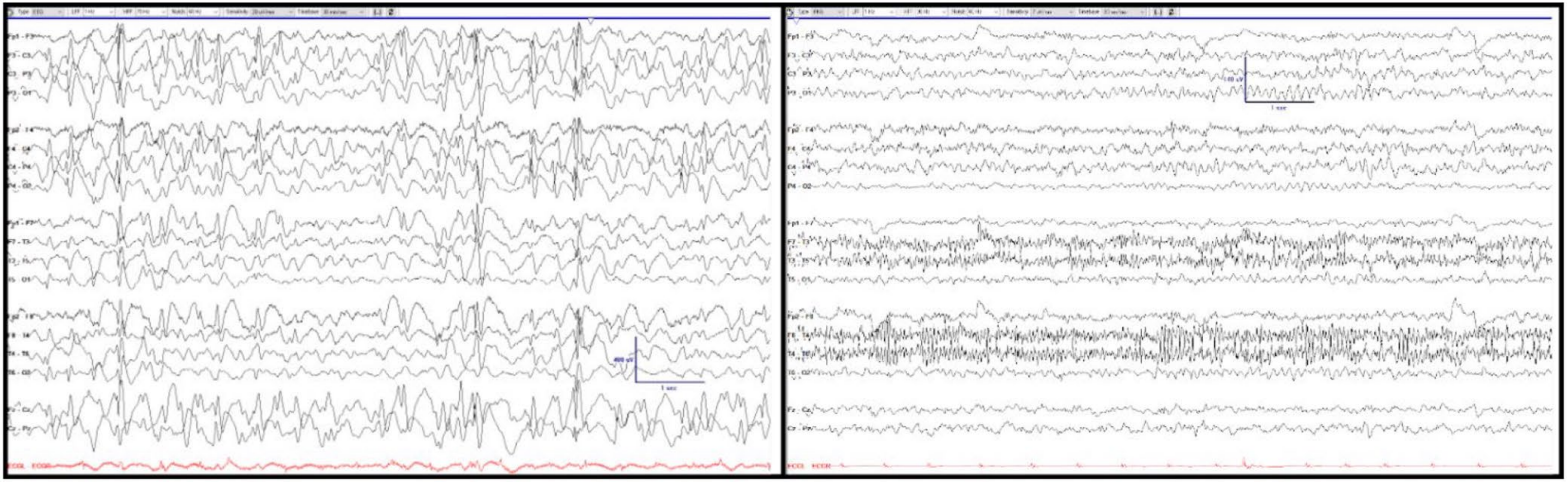
Longitudinal bipolar EEG recordings before and after treatment with valproic acid.

This case illustrates a rare presentation of LGS secondary to a pericallosal lipoma, with a remarkable EEG and clinical response to valproic acid. Intracranial lipomas are rare findings on imaging.^1^ Although typically asymptomatic, they can present with neurologic symptoms depending on their size and location. A pericallosal lipoma may disrupt corpus callosal function and lower the seizure threshold through interhemispheric disconnection, contributing to seizure activity.^2^, ^3^, ^4^ However, no cases of LGS secondary to pericallosal lipoma have been reported before. Structural brain abnormalities account for approximately 40% of LGS cases.^5^ Valproic acid remains a cornerstone in managing LGS and was particularly effective in this case, especially when surgery is a less preferable option. These findings underscore the need for further investigation into the pathophysiologic mechanisms linking structural anomalies with epileptic encephalopathies like LGS.

Informed consent was obtained from the parent of the patient presented prior to beginning this work.

## AUTHOR CONTRIBUTIONS

Conception and design: RMLLR, HLC, and DN. Data collection: RMLLR and DN. Data analysis and interpretation: RMLLR, HLC, and DN. Manuscript drafting: RMLLR and HLC. Figures preparation: RMLLR and DN. Critical revision of manuscript: RMLLR, HLC, and DN.

## FUNDING INFORMATION

No funding was received.

## CONFLICT OF INTEREST STATEMENT

The authors declare no conflict of interest.

**Test yourself**

1. Which of the following best explains the proposed mechanism by which a pericallosal lipoma may contribute to epilepsy?
  - Direct secretion of pro‑convulsant lipokines into the CSF
  - Disruption of interhemispheric inhibitory and facilitatory functions of the corpus callosum
  - Compression of the basal ganglia leading to dopaminergic overactivity
  - Secondary autoimmune encephalitis targeting meningeal fat cells
2. In a child with Lennox–Gastaut syndrome and a known pericallosal lipoma, which MRI feature most strongly supports that the lipoma is a congenital malformation typical of intracranial lipomas rather than an acquired lesion?
  - Irregular ring enhancement and surrounding vasogenic edema after contrast
  - Homogenous signal identical to fat in all sequences with suppression on fat‐saturated images
  - Restricted diffusion within the lesion on diffusion‐weighted imaging
  - Progressive enlargement over months with marked mass effect on ventricles
3. Regarding intracranial lipomas as a cause of epilepsy, which management approach is generally most appropriate in a child with Lennox–Gastaut syndrome and a classic, nonmass‐effect pericallosal lipoma?
  - Conservative management focusing on optimal antiseizure medications and syndrome‐directed therapies
  - Aggressive surgical resection of the lipoma as first‐line therapy
  - High‐dose corticosteroids to decrease perilesional edema and control seizures
  - Immediate cranial radiotherapy to reduce the size of the lipoma

*Answers may be found in the* *supporting information*.

## Supporting information


Data S1.

Data S2.

## Data Availability

Data were shared in the manuscript. No dataset was generated for this study. This study did not reproduce material from other sources.
