# Supplementary material for: A rare pericallosal lipoma presenting with Lennox–Gastaut syndrome and a remarkable response to valproic acid
Source: Epileptic Disord. 2026 Apr 24;28(4):1313–5. doi: 10.1002/epd2.70249 (PMC13499244; doi:10.1002/epd2.70249)
Supplement: Supplementary file 1 [file EPD2-28-1313-s001.docx]

**Test Yourself Answers**

1. Correct answer: B

Explanation: Corpus callosal/pericallosal lipomas can interfere with normal interhemispheric connectivity, reducing the corpus callosum's inhibitory influence and lowering seizure threshold.

2. Correct answer: B

Explanation: Intracranial lipomas, including pericallosal lipomas, characteristically demonstrate imaging features identical to mature adipose tissue. On MRI, they appear hyperintense on T1-weighted images and variably hyperintense on T2-weighted images, and they fully suppress on fat-saturated or short-tau inversion recovery sequences.

3. Correct answer: A

Explanation: For most intracranial lipomas, particularly pericallosal lipomas without mass effect or progressive enlargement, the best management strategy is conservative. These lesions are congenital, stable, and tightly associated with critical neurovascular structures, making them poor surgical targets.
